# Age-dependent diffusion-relaxation coupling in the basal ganglia: Implications for iron deposition and microstructural dynamics

**DOI:** 10.1162/IMAG.a.1195

**Published:** 2026-04-02

**Authors:** Kaibao Sun, Yezhe Wang, Xiaoyue Sun, Youjia Zhang, Xiaohong Joe Zhou, Hesheng Liu

**Affiliations:** Changping Laboratory, Beijing, China; Center for Magnetic Resonance Research and Department of Radiology, University of Illinois Chicago, Chicago, IL, United States; Biomedical Pioneering Innovation Center, Peking University, Beijing, China

**Keywords:** diffusion-relaxation coupling, aging, iron deposition, tissue microstructures

## Abstract

This study investigates age-related changes in diffusion-relaxation coupling within basal ganglia nuclei using a novel single-scan multi-TE diffusion-weighted imaging (SMT-DWI) approach, with a focus on microstructural alterations associated with regional iron deposition patterns during normal brain aging. Fifty-seven healthy participants (10–73 years) underwent optimized SMT-DWI at 3T, simultaneously acquiring multi-b-value (0/500/1000 s/mm²) and multi-TE (53/71/89 ms) data in 7:58 minutes (1.5 × 1.5 × 4 mm³ resolution). Apparent diffusion coefficient (ADC) and R2 maps were generated through mono-exponential fitting, with coupling quantified via TE-dependent ADC (k_ADC/TE_) and b-value-dependent R2 (k_R2/b_) metrics. Iron-related tissue properties were assessed using R2 at b = 0 (R2_b = 0_) as a surrogate. The SMT-DWI method achieved excellent image quality (e.g., SNR = 63.8 under the most unfavorable conditions of b = 1000 s/mm² and TE = 89 ms) with good anatomical delineation. Young subjects exhibited positive coupling (ADC increasing with TE, R2 rising with b-value) across basal ganglia, while older adults showed progressive inversion to negative coupling that correlated strongly with age after accounting for sex as a covariant (sex was not a significant predictor; all p > 0.05). This transition was the most pronounced in iron-rich putamen (p < 0.001, R2_b = 0_ = 0.0208 ± 0.0027 ms^−^¹), globus pallidus (p < 0.001, R2_b = 0_ = 0.0276 ± 0.0030 ms^−^¹), and substantia nigra (p < 0.001, R2_b = 0_ = 0.0241 ± 0.0023 ms^−^¹), while the caudate maintained stable coupling (p > 0.05) with lower iron levels (R2_b = 0_ = 0.0169 ± 0.0012 ms^−^¹). All four regions demonstrated significant negative correlations between coupling metrics and R2_b = 0_ (r = -0.38 to -0.75, all p < 0.01), consistent with a role for iron-mediated microstructural changes in driving the observed coupling shifts. The age-dependent inversion from positive to negative diffusion-relaxation coupling reflects regionally heterogeneous microstructural alterations in basal ganglia. Our method provides sensitive detection of compartment-sensitive microstructural alterations, offering new insights into normal brain aging and a potential biomarker for neurodegenerative risk assessment.

## 1 Introduction

The human brain demonstrates remarkable neuroplasticity, with its microstructure and neural networks undergoing continuous remodeling throughout ontogeny (Sowell et al., 2003). Within this framework, the basal ganglia (comprising the caudate nucleus, putamen, globus pallidus, and substantia nigra)—crucial regulators of motor function, cognitive processing, and affective states—exhibit particularly dynamic trajectories of age-related change (Alexander, 1986; Chakravarthy et al., 2010; Gold et al., 2005; Rodriguez-Sabate et al., 2022). These nuclei are composed of heterogeneous tissue components, primarily glial cells, neuronal cell bodies, and axonal tracts, which exhibit distinct biophysical properties (Haber, 2016). Magnetic resonance imaging (MRI) techniques, particularly diffusion-weighted imaging (DWI) and transverse relaxation rate (R2, i.e., 1/T2) mapping, offer powerful tools for probing these microstructures *in vivo* (Chatterjee et al., 2018; Does & Gore, 2000; Ning et al., 2020; Slator et al., 2021; S. Wang et al., 2014). While DWI quantifies water diffusion patterns sensitive to tissue organization (Beaulieu, 2002), R2 mapping reflects local magnetic susceptibility and water content (Yablonskiy & Haacke, 1994). Together, they provide complementary insights into tissue microstructure, enabling the investigation of diffusion-relaxation coupling (Sun et al., 2023)—a phenomenon where the apparent diffusion coefficient (ADC) and R2 exhibit interdependent behavior driven by compartment-sensitive restrictions and molecular interactions.

In the basal ganglia, neuronal cell bodies typically exhibit higher ADC and lower R2 values due to their larger size and higher water content, while axonal tracts show lower ADC and higher R2 values due to their dense, anisotropic structure (Helenius et al., 2002; Sener, 2001; Stanisz et al., 2005; Yoshiura et al., 2001). This intrinsic contrast gives rise to predictable patterns of diffusion-relaxation coupling, which can be quantified using advanced MRI techniques (Chatterjee et al., 2018; Does & Gore, 2000; Ning et al., 2020; Slator et al., 2021; S. Wang et al., 2014). Notably, the aging process and neurodegenerative diseases are often accompanied by significant alterations in tissue microstructure, including the accumulation of iron in the basal ganglia (Angelova & Brown, 2015; Ward et al., 2014; Zecca et al., 2004). Iron is a paramagnetic substance that increases the R2 relaxation rate and may have indirect effects on water diffusion (Haacke et al., 2005; Pfefferbaum et al., 2008), suggesting an alternative coupling mechanism between diffusion and relaxation properties in iron-rich regions. This decoupling may provide a novel biomarker for microstructural alterations related to iron deposition, aging, and neurodegeneration.

Iron accumulation in the basal ganglia is a hallmark of both normal aging and neurodegenerative disorders, including Parkinson’s, Alzheimer’s and Huntington’s diseases (Zecca et al., 2004). While physiological iron supports cellular metabolism and neurotransmitter synthesis (Beard, 2003), excessive deposition induces oxidative stress and neuroinflammation, driving neuronal damage (Kell, 2009). Conventional MRI methods for assessing iron content, such as R2* (1/T2*) (Langkammer et al., 2010), susceptibility-weighted imaging (SWI) (Haacke et al., 2004), or quantitative susceptibility mapping (QSM) (Haacke et al., 2015; Y. Wang & Liu, 2015), lack specificity for cellular compartments. In contrast, diffusion-relaxation coupling analysis offers a unique advantage: its sensitivity to contributions from cellular compartments (e.g., cell bodies vs. axons) may reveal how iron and other age-related microstructural changes alter specific tissue microenvironments rather than merely reporting bulk susceptibility changes.

Emerging studies have leveraged diffusion-relaxation coupling to characterize tissues such as the prostate (Chatterjee et al., 2018; S. Wang et al., 2014), placenta (Slator et al., 2019, 2023), and brain (Johansson et al., 2024), revealing compartment-sensitive signatures. For instance, in the prostate, positive diffusion-relaxation coupling [ADC increasing with echo time (TE); R2 rising with b-value] has been observed and has been used to quantify the component fraction of epithelium, stroma, and lumen (Chatterjee et al., 2018; S. Wang et al., 2014). In the healthy brain and brain tumors, white matter exhibits complex ADC-R2 interdependencies modulated by intra- and extra-axonal water compartmentation (Johansson et al., 2024). However, the behavior of diffusion-relaxation coupling in the basal ganglia, particularly in the context of aging and microstructural alterations potentially linked to iron deposition, remains poorly understood. This knowledge gap critically limits both the interpretation of MRI data and the development of new biomarkers for brain aging and neurodegeneration.

This study investigates lifespan trajectories of diffusion-relaxation coupling in the basal ganglia, with a focus on microstructural alterations. We hypothesize that age-related microstructural changes, including but not limited to iron deposition, differentially affect neuronal and axonal compartments, manifesting as quantifiable coupling patterns. By integrating multi-b-value DWI and multi-echo R2 mapping into a single-scan protocol, our study aims at establishing a clinically feasible framework for characterizing tissue microstructures.

## 2 Methods

### 2.1 Participants

The study was conducted after the institutional review board approval. With written informed consent, 57 healthy participants (24 male; age range: 10–73 years) were enrolled between January 2025 and May 2025. The age distribution was relatively balanced across decades (10–19: n = 8, 20–29: n = 15, 30–39: n = 11, 40–49: n = 12, 50–69: n = 10, 70–79: n = 1). Exclusion criteria comprised MRI contraindications, neurological disorders, severe basal ganglia calcification, or structural brain abnormalities.

### 2.2 Diffusion-relaxation coupling model

To provide a conceptual framework for understanding diffusion-relaxation coupling in heterogeneous tissues, we employed a biophysical framework proposed by Sun et al. (2023), which describes signal contributions from two or more tissue compartments with distinct diffusion and relaxation properties. Consider a specific tissue with two components having different diffusion properties and R2 relaxation rates. The composite signal intensity S(TE, b) that takes into account of contributions from both components can be expressed as: $\text{S}\left(\text{TE},\text{ b}\right)/{\text{S}}_{0}=$ ${\text{f}}_{1}\exp\left(-\text{R}2_{1}\times \text{TE}\right)$ $\exp\left(-{\text{ADC}}_{1}\times \text{b}\right)+{\text{f}}_{2}\exp\left(-\text{R}2_{2}\times \text{TE}\right)\exp$ $\left(-{\text{ADC}}_{2}\times \text{b}\right)$, where ${\text{f}}_{1}$ and ${\text{f}}_{2}$ are the volume fractions of the two components, each with its own ADC and R2 values as indicated by the subscripts; and ${\text{S}}_{0}$ is the signal intensity when TE = 0 ms and b-value = 0 s/mm^2^. This bi-exponential model serves as a phenomenological framework to illustrate the possible origin of diffusion-relaxation coupling, rather than as a computational model for direct data fitting. The coupling phenomenon arises when distinct tissue compartments exhibit differences in both ADCs and R2s. Specifically, positive diffusion-relaxation coupling is defined when (ADC_1_ − ADC_2_) × (R2_1_ − R2_2_) < 0. In this case, prolonging the TE preferentially attenuates signals from the higher-R2 compartment, thereby increasing the measured voxel-wise ADC due to the preservation of signals from the lower-R2 (typically higher-ADC) compartment. Similarly, increasing the diffusion weighting (b-value) suppresses signals from the higher-ADC compartment, resulting in an increase in the measured voxel-wise R2. Conversely, negative diffusion-relaxation coupling occurs when (ADC_1_ − ADC_2_) × (R2_1_ − R2_2_) > 0. Here, longer TEs lead to decreased ADC measurements as the higher-R2 (now higher-ADC) compartment becomes attenuated, while higher b-values decrease the observed R2 due to selective suppression of the higher-ADC (higher-R2) compartment. This model conceptually enables the quantification of diffusion-relaxation coupling through examination of TE-dependent ADC and b-value-dependent R2 variations.

### 2.3 Single-scan multi-TE diffusion-weighted imaging (SMT-DWI)

**Fig. 1.**
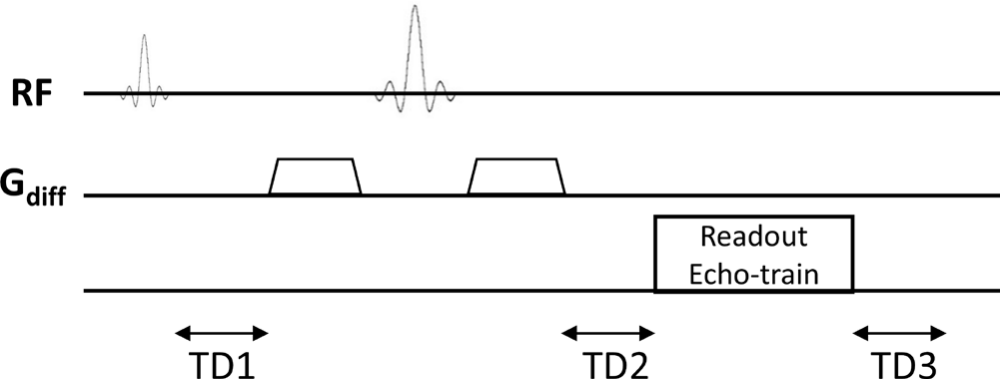
Schematic diagram of the single-scan multi-echo diffusion-weighted imaging (SMT-DWI) pulse sequence. The sequence employs a time-optimized diffusion encoding scheme with fixed diffusion gradient timing and adjustable symmetric delays (TD1/TD2) flanking the diffusion-encoding gradients, enabling flexible echo time (TE) modulation while maintaining consistent b-values. To ensure constant repetition time across different TEs, the post-readout delay (TD3) is dynamically adjusted in inverse proportion to TD1/TD2 variations. To achieve trace-weighting, diffusion gradients were applied sequentially along the three orthogonal axes (X, Y, or Z) in separate acquisitions; the geometric mean of these three directionally encoded images yields the final trace-weighted image for each (TE, b-value) combination. This design permits acquisition of diffusion-weighted images at multiple TEs within a single scan session, thereby minimizing the impact of inter-scan motion while ensuring identical geometric distortion patterns in the phase-encoding direction across all TE images.

A custom single-scan multi-TE diffusion-weighted imaging (SMT-DWI) pulse sequence was developed to acquire diffusion-weighted images at multiple echo times within a single scan session (Fig. 1). Central to the pulse sequence was a time-optimized diffusion encoding scheme that minimized the diffusion gradient duration while enabling flexible TE adjustments. To achieve this, the shortest possible time interval for diffusion encoding (Δ = 19.9 ms, δ = 9.5 ms) was implemented, and different TEs were realized by symmetrically inserting adjustable delays (0–18 ms) before and after the diffusion-encoding gradient pair. This approach maintained consistent diffusion weighting (b-values: 0, 500, 1000 s/mm²) across all TEs while allowing TE to vary between 53 ms (limited by the gradient system and/or the highest b-value) and 89 ms. Our trace-weighted images were derived from sequential, single-direction acquisitions along the three orthogonal axes (X, Y, or Z) one at a time, and the geometric mean of these three separate acquisitions was computed. This sequential method avoids the complex cross-term concomitant fields that arise from simultaneous multi-axis encoding (Du et al., 2002; Finkelstein et al., 2022; Mori & Zijl, 1995; Wong et al., 1995; X. J. Zhou, Du, et al., 1998; X. J. Zhou, Tan, et al., 1998). By integrating multiple TEs into a single scan, the SMT-DWI sequence aimed to reduce inter-scan motion artifacts inherent to conventional multi-run acquisitions. This design enabled precise voxel-wise analysis of ADC mapping and R2 mapping for diffusion-relaxation coupling quantification with improved spatial consistency across TEs.

### 2.4 Image acquisition

The SMT-DWI sequence was implemented and evaluated on a GE signa UHP 3.0 T scanner (GE Healthcare, Waukesha, Wisconsin, USA) with a maximum gradient strength of 100 mT/m and a maximum slew rate of 200 T/m/s. All participants were imaged with a 48-channel phased-array head coil (GE Healthcare, Waukesha, Wisconsin, USA). The imaging protocol included high-spatial-resolution sagittal T1-weighted imaging with full brain coverage and SMT-DWI with three b values and three TEs covering the basal ganglia, which resulted in a 3 × 3 array of data associated with each voxel. At each nonzero b-value, the diffusion gradient was applied successively along the three orthogonal directions to obtain a trace-weighted image to mitigate the effects of diffusion anisotropy. The other key acquisition parameters of SMT-DWI were: TR = 3500 ms, FOV = 192 × 192 mm^2^, imaging matrix = 128 × 128, number of slices = 20, slice thickness = 4 mm, slice spacing = 0.5 mm, and [TE(ms), b value(s/mm^2^)]_NEX (number of excitations)_ = [53, 0]_3_/[71, 0]_3_/[89, 0]_3_/[53, 500]_2_/[71, 500]_4_/[89, 500]_8_/[53, 1000]_4_/[71, 1000]_8_/[89, 1000]_16_, and the scan time = 7 minutes and 58 seconds.

### 2.5 Image analysis

Diffusion-weighted images acquired at the three b-values and the three TEs were individually reconstructed. Quantitative measurement of the signal-to-noise ratio (SNR) was performed on the image with the highest b-value (i.e., 1000 s/mm^2^) and the longest TE (i.e., 89 ms), where the signal was maximally attenuated to assess the worst-case scenario. Prior to quantitative analysis, diffusion-weighted images for each TE and b-value combination were coregistered to their respective reference image (b = 0 s/mm², TE = 53 ms) using a rigid-body registration algorithm implemented in FSL (FMRIB Software Library) (Andersson & Sotiropoulos, 2016) to correct for intra-scan motion.

All images with different b-values and TEs were analyzed using custom MATLAB (The MathWorks, Inc., Natick, MA, USA) programs. Voxel-wise ADCs were computed for each TE from signals acquired with the three b-values. Similarly, voxel-wise R2 values at each b-value were calculated from signals acquired with the three TEs. Both ADC and R2 quantifications were performed using non-linear least-squares fitting of the mono-exponential signal model.

For each participant, regions of interest (ROIs) were drawn bilaterally on the hypointense region of the basal ganglia in the diffusion-weighted images (b = 500 s/mm^2^, TE = 71 ms). The ROI selections were performed by K.S. under the guidance of two radiologists, each having more than 10 years of experience. The structure of the basal ganglia was identified in at least three contiguous sections, and the middle section was chosen to draw the regions of interest to minimize partial-volume effects.

To quantify age-related changes in diffusion-relaxation coupling in basal ganglia nuclei, we performed ROI-based analyses of the ADC and R2 relaxation rate across multiple TEs and b-values, respectively. For each predefined ROIs, including the caudate, putamen, globus pallidus, and substantia nigra, the analysis proceeded as follows. Mean ADC and R2 values were computed by averaging all voxels within each ROI for each TE (53, 71, 89 ms) and each b-value (0, 500, 1000 s/mm²), respectively. The mean ADC values for each ROI were linearly regressed against TE. The slope of this regression (k_ADC/TE_, defined as the TE-dependence of ADC) was calculated to quantify the TE dependence on ADC, defined as the TE-ADC coupling metric. Similarly, the mean R2 values were linearly regressed against b-value. The slope of this regression (k_R2/b_, defined as the b-value-dependence of R2) was computed to assess the b-value dependence on R2, termed the b-R2 coupling metric. These directional metrics provide experimental evidence of the theoretical coupling relationships: positive coupling corresponds to k_ADC/TE_ > 0 and k_R2/b_ > 0, while negative coupling shows the inverse relationships.

### 2.6 Statistical analysis

Statistical analyses were performed by using MATLAB toolbox. To assess age-related trends, multiple linear regression was performed between the coupling metrics and participant age across all subjects, with sex included as a covariate (model: coupling_metric = β_0_ + β_1_ × age + β_2_ × sex). We also evaluated a model that included a quadratic age term (age²) alongside the linear age term and sex covariate. However, the quadratic term did not reach statistical significance in any region after correction for multiple comparisons, nor did it improve model fit; therefore, we retained the simpler linear model with sex as a covariate for final reporting to enhance interpretability. To assess whether alterations in coupling were mediated by microstructural changes associated with iron deposition, we correlated coupling metrics with R2 values at b = 0 (R2_b = 0_), which served as a surrogate for tissue properties including iron content. While R2* weighted imaging is conventionally used for iron quantification, R2_b = 0_ was chosen due to its spatial consistency with coupling data, ensuring identical geometric distortions and eliminating registration errors. Prior studies have demonstrated strong positive correlations between R2 and iron deposition in iron-rich regions (Langkammer et al., 2010), though it is acknowledged that R2 is also influenced by other factors such as myelin content, supporting its validity in this context. Correction for multiple comparisons across all correlation analyses was performed using the False Discovery Rate (FDR) method, and the FDR-adjusted p-values are reported where applicable.

## 3 Results

**Fig. 2.**
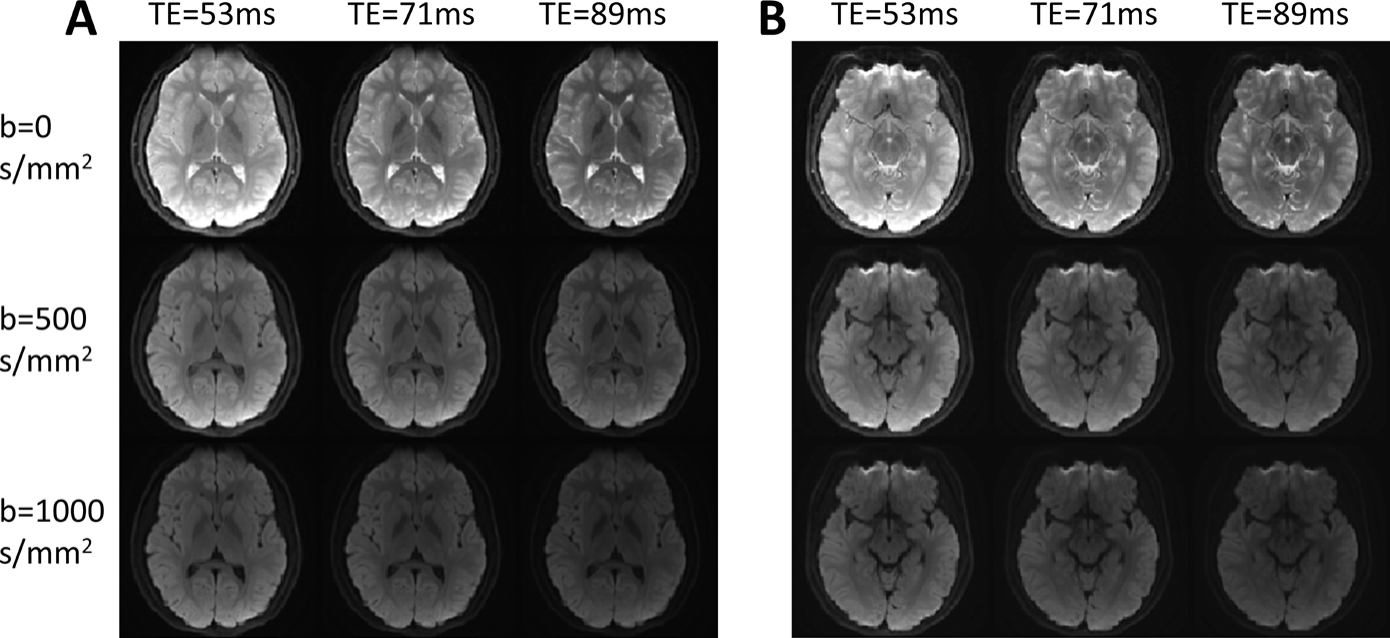
Representative single-scan multi-TE diffusion-weighted images of the basal ganglia from a representative healthy subject (12 years old) acquired using the SMT-DWI sequence, displayed in a 3 × 3 matrix showing all combinations of three b-values (0, 500, 1000 s/mm²) and three echo times (53, 71, 89 ms). (A) An oblique axial section through the dorsal striatum displays the caudate, putamen, and globus pallidus. (B) Inferior oblique axial section shows the substantia nigra. All images were acquired in a single scan session with identical geometric parameters. Signal intensity variations reflect the coupled effects of diffusion restriction and R2 decay, enabling voxel-wise studies of diffusion-relaxation coupling.

The SMT-DWI sequence successfully acquired diffusion-weighted images at three TEs (53, 71, and 89 ms) in a single scan session lasting 7 minutes and 58 seconds, eliminating potential misregistration artifacts inherent in sequential acquisitions while maintaining consistent spatial resolution (1.5 × 1.5 × 4 mm³). Figure 2 presents two representative oblique axial slices from a 12-year-old participant, demonstrating accurate spatial co-registration after motion correction without motion-related contamination. The 3 × 3 image matrices display the interplay between diffusion weighting (b-values: 0, 500, 1000 s/mm² along the vertical axis) and relaxation effects (TEs: 53, 71, 89 ms along the horizontal axis), providing comprehensive visualization of contrast evolution. Quantitative analysis confirmed excellent SNR (SNR = 63.8) even under the most unfavorable conditions (b = 1000 s/mm², TE = 89 ms).

**Fig. 3.**
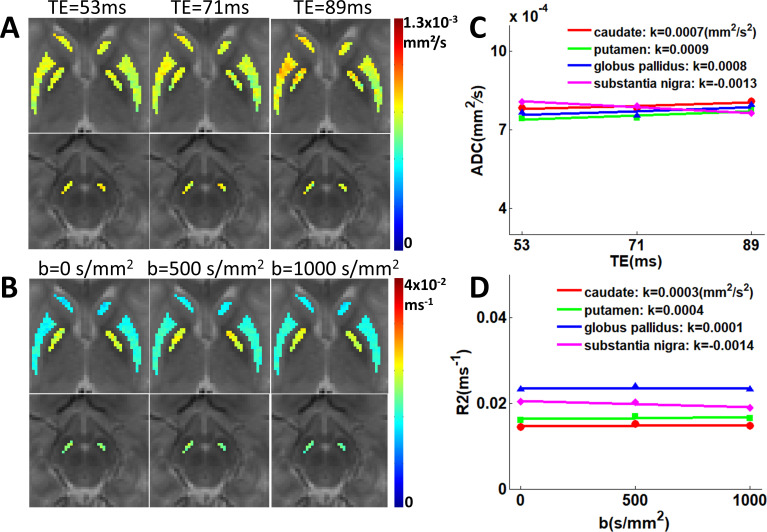
Representative diffusion-relaxation coupling in a young subject, shown in Figure 2. (A) ADC maps of the caudate, putamen, globus pallidus, and substantia nigra at different echo times (TEs: 53, 71, 89 ms) and (B) R2 maps at various b-values (0, 500, 1000 s/mm²) are overlaid on an image with a b-value of 0 s/mm² and TE of 53 ms. (C) Quantitative plots of mean ADC values versus TE across basal ganglia subregions, with the following rates of change: k_ADC/TE_ (mm²/s²) =0.0007 (caudate), 0.0009 (putamen), 0.0008 (globus pallidus), and -0.0013 (substantia nigra). (D) Mean R2 values versus b-value, demonstrating corresponding changes: k_R2/b_ (mm²/s²) = 0.0003 (caudate), 0.0004 (putamen), 0.0001 (globus pallidus), and -0.0014 (substantia nigra).

Figure 3A and B presents the derived parametric maps, with ADC maps demonstrating TE dependence and R2 maps showing b-value dependence. The quantitative plots of mean ADC and R2 values within each ROI are shown in Figure 3C and D for each TE (53, 71, 89 ms) and b-value (0, 500, 1000 s/mm²), respectively. ADC values increased with longer TEs in most basal ganglia regions (k_ADC/TE_ values of 0.0007, 0.0009, and 0.0008 mm²/s² for caudate, putamen, and globus pallidus respectively), while R2 values showed corresponding increases with higher b-values (k_R2/b_ values of 0.0003, 0.0004, and 0.0001 mm²/s² for the same regions), demonstrating a predominant pattern of positive diffusion-relaxation coupling in the basal ganglia of the youth. An exception was observed in the substantia nigra, which showed clear negative coupling (k_ADC/TE_ = -0.0013 mm²/s², k_R2/b_ = -0.0014 mm²/s²).

**Fig. 4.**
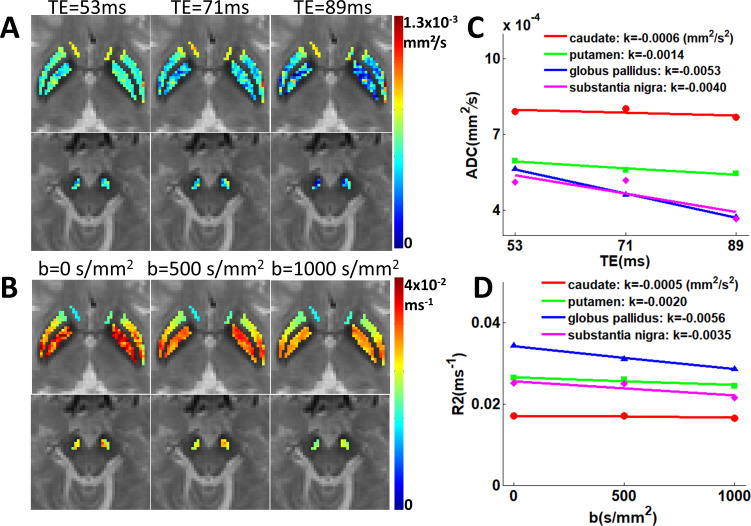
Negative diffusion-relaxation coupling in an older subject. (A) ADC maps and (B) R2 maps of the caudate, putamen, globus pallidus, and substantia nigra in a representative 55-year-old subject, highlighting distinct contrast dependencies compared to Figure 3. (C) ADC values decreasing with TE (k_ADC/TE_ = -0.0006 to -0.0053). (D) R2 values decreasing with b-value (k_R2/b_ = -0.0005 to -0.0056) across the basal ganglia nuclei.

**Fig. 5.**
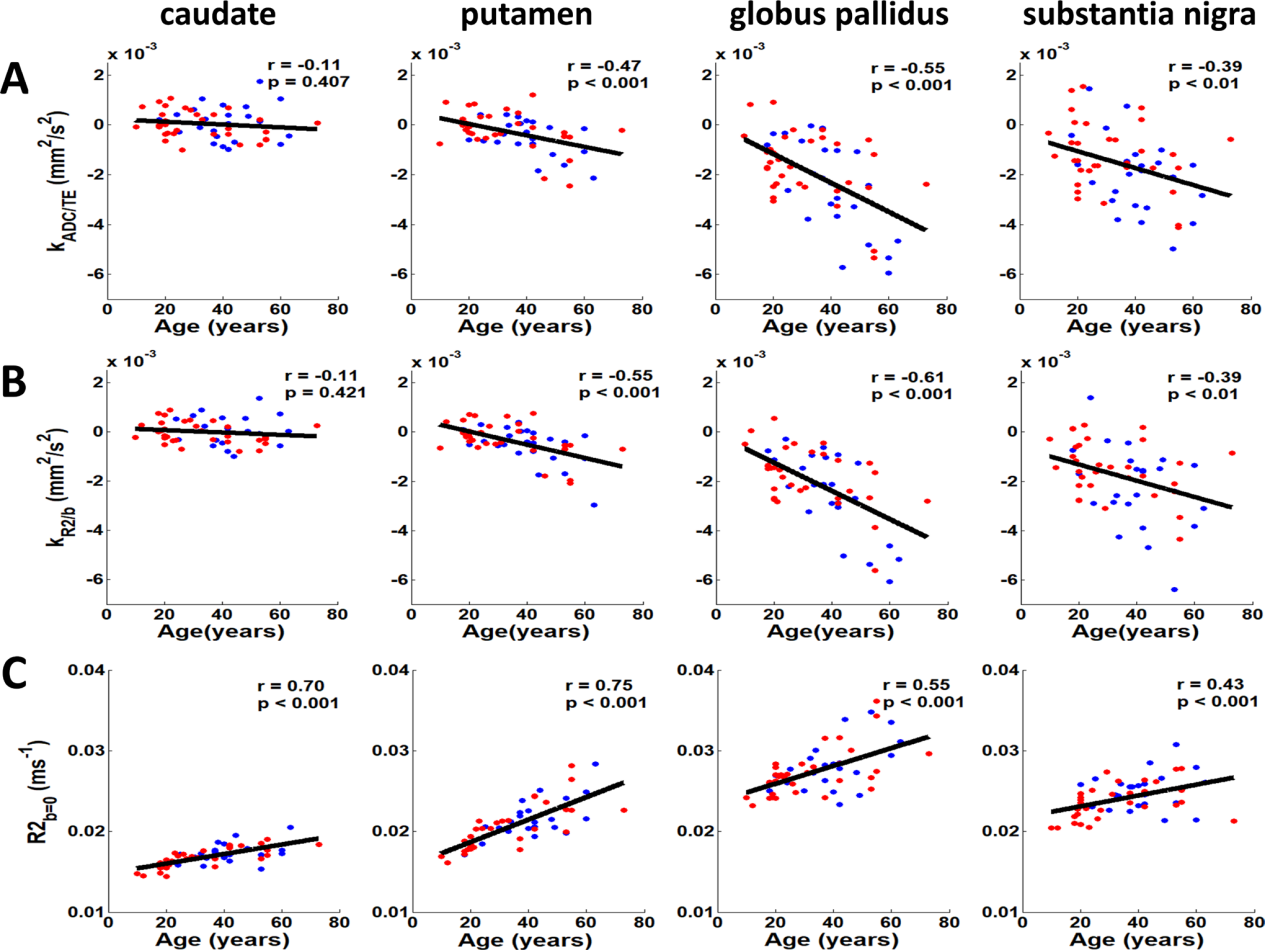
Population-level age correlations of coupling metrics and R2_b = 0_. Scatter plots illustrate age associations, with male and female participants denoted by blue and red markers, respectively. The regression line reflects the group-level fit performed on the combined cohort, as sex was not a significant predictor in any model (all p > 0.05). (A) k_ADC/TE_ and (B) k_R2/b_ in the putamen (k_ADC/TE_: r = -0.47; k_R2/b_: r = -0.55), globus pallidus (k_ADC/TE_: r = -0.55; k_R2/b_: r = -0.61), and substantia nigra (k_ADC/TE_: r = -0.39; k_R2/b_: r = -0.39). The caudate shows no significant age dependence (p > 0.05). (C) Mean R2 derived from b = 0 images across TEs (R2_b = 0_) shows significant positive correlations with age in all four regions (p < 0.001). All reported p-values are corrected for multiple comparisons across analyses using the False Discovery Rate (FDR) method.

A different coupling pattern emerged in elderly participants, as illustrated by data from a representative 55-year-old subject (Fig. 4). Here, we observed characteristic negative coupling, with ADC values decreasing with TE (k_ADC/TE_ = -0.0006, -0.0014, -0.0053, and -0.0040 mm^2^/s^2^ for caudate, putamen, globus pallidus, and substantia nigra respectively) and R2 values decreasing with higher b-values (k_R2/b_ = -0.0005, -0.0020, -0.0056, and -0.0035 mm^2^/s^2^ for the same regions). Population-level analysis on all 57 healthy subjects confirmed this age-dependent shift of coupling dynamics. First, we confirmed that sex was not a significant predictor of the coupling metrics in any region (all p > 0.05). We subsequently performed the primary correlation analysis on the combined cohort, which revealed statistically significant negative correlations between coupling metrics and age in the putamen (k_ADC/TE_: r = -0.47, p < 0.001; k_R2/b_: r = -0.55, p < 0.001), globus pallidus (k_ADC/TE_: r = -0.55, p < 0.001; k_R2/b_: r = -0.61, p < 0.001), and substantia nigra (k_ADC/TE_: r = -0.39, p < 0.01; k_R2/b_: r = -0.39, p < 0.01). Notably, the caudate showed no significant age-related changes in coupling metrics (k_ADC/TE_: r = -0.11, p > 0.05; k_R2/b_: r = -0.11, p > 0.05), which was likely due to minimal microstructural change of ADC and/or R2 (Fig. 5A, B). In contrast, R2_b = 0_ showed significant positive correlations with age across all four regions, including the caudate (all p < 0.001) (Fig. 5C).

**Fig. 6.**
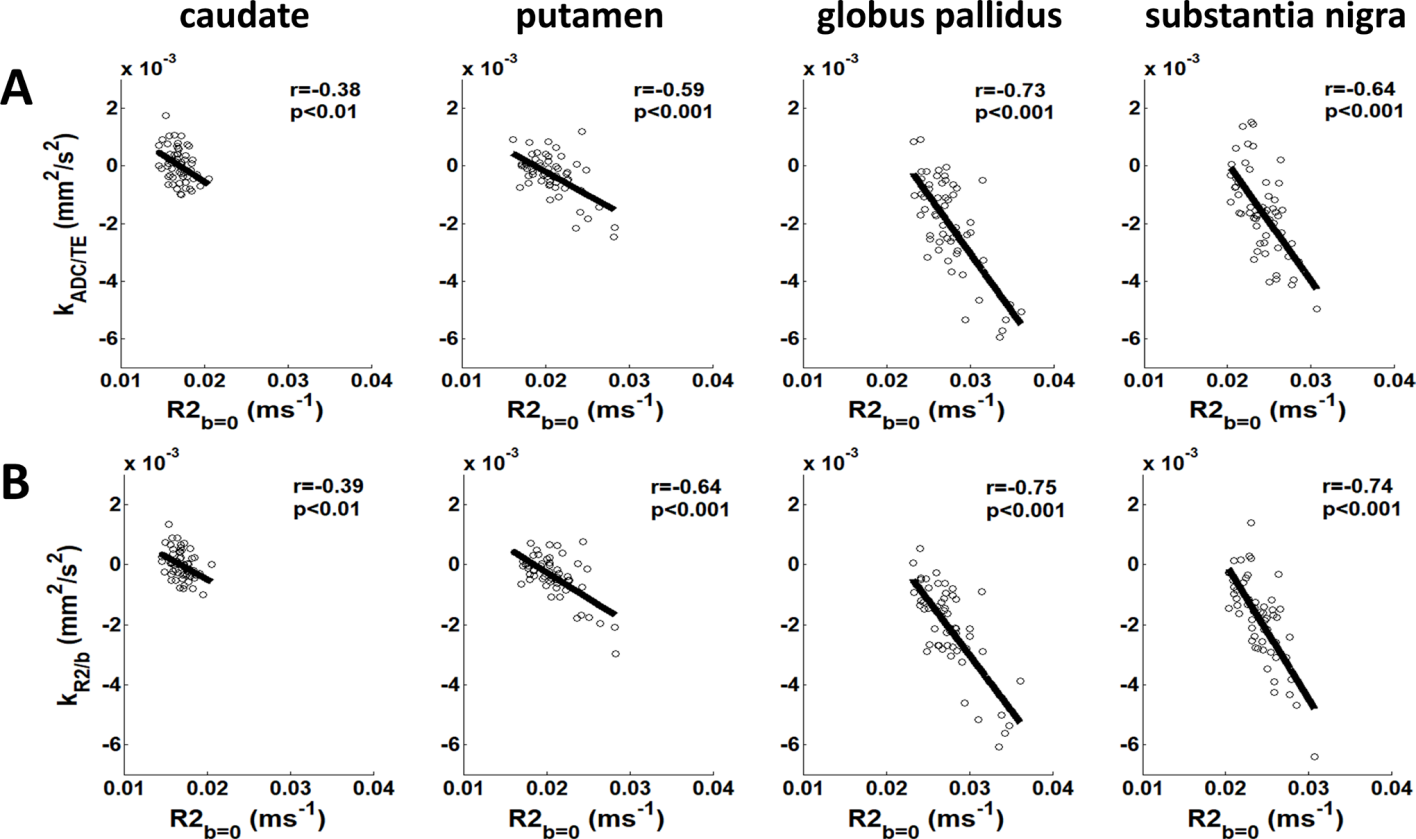
Iron-driven diffusion-relaxation coupling changes. Regional R2_b = 0_ values along the horizontal axis demonstrate the hierarchy of tissue properties, consistent with known patterns of iron accumulation. Significant negative correlations between coupling metrics (A: k_ADC/TE_; B: k_R2/b_) and R2_b = 0_ were found in the caudate (k_ADC/TE_: r = -0.38; k_R2/b_: r = -0.39), putamen (k_ADC/TE_: r = -0.59; k_R2/b_: r = -0.64), globus pallidus (k_ADC/TE_: r = -0.73; k_R2/b_: r = -0.75), and substantia nigra (k_ADC/TE_: r = -0.64; k_R2/b_: r = -0.74). All reported p-values are corrected for multiple comparisons across analyses using the False Discovery Rate (FDR) method.

The observed coupling shifts strongly correlated with tissue properties indexed by R2_b = 0_ (Fig. 6). Regional R2_b = 0_ measurements revealed expected patterns of iron accumulation, with the highest values in the globus pallidus (0.0276 ± 0.0030 ms^−^¹) and substantia nigra (0.0241 ± 0.0023 ms^−^¹), intermediate levels in the putamen (0.0208 ± 0.0027 ms^−^¹), and lowest in the caudate (0.0169 ± 0.0012 ms^−^¹) consistent with its minimal iron accumulation. As demonstrated in Figure 6, coupling metrics showed significant negative correlations with R2_b = 0_ across all iron-sensitive regions, including caudate (k_ADC/TE_: r = -0.38, p < 0.01; k_R2/b_: r = -0.39, p < 0.01), putamen (k_ADC/TE_: r = -0.59, p < 0.001; k_R2/b_: r = -0.64, p < 0.001), globus pallidus (k_ADC/TE_: r = -0.73, p < 0.001; k_R2/b_: r = -0.75, p < 0.001), and substantia nigra (k_ADC/TE_: r = -0.64, p < 0.001; k_R2/b_: r = -0.74, p < 0.001). These findings implicated iron accumulation as the primary driver of age-related changes in diffusion-relaxation coupling dynamics.

## 4 Discussion

Our study reveals an age-dependent reversal of diffusion-relaxation coupling in basal ganglia nuclei, transitioning from positive coupling in young individuals to negative coupling in elderly adults. This coupling shift strongly correlates with region-specific iron accumulation, suggesting a potential mechanistic link between altered microstructure and iron deposition during aging. Our SMT-DWI protocol enabled measurement of this phenomenon through simultaneous multi-parametric mapping, achieving sufficient SNR while avoiding motion-induced misregistration artifacts. The approach with SMT-DWI offers complementary information to conventional iron-sensitive techniques by probing compartment-sensitive microstructural changes.

The observed positive coupling in young subjects aligns with the known biophysical properties of neuronal cell bodies and axonal tracts. Neuronal cell bodies, with their larger size and higher water content, dominate the diffusion and relaxation properties of healthy tissue, leading to increased ADC with TE and rising R2 with b-value. This pattern serves as a baseline for understanding the microstructure of basal ganglia in the absence of excessive iron deposition. These findings are supported by previous studies showing that neuronal cell bodies exhibit lower R2 relaxation rates and higher ADC values compared to axonal tracts, which are more densely packed and anisotropic (Helenius et al., 2002; Sener, 2001; Stanisz et al., 2005; Yoshiura et al., 2001) (i.e., (ADC_cellbody_ − ADC_axon_) × (R2_cellbody_ − R2_axon_) < 0).

The shift to negative coupling in elderly subjects likely reflects the compartment-sensitive biophysical consequences of age-related alterations, prominently including iron accumulation. Previous studies show that iron primarily deposits in the cell bodies (Connor et al., 1990; Ke & Qian, 2007; Moos et al., 2007), where it increases R2 relaxation rate but has minimal effects on water diffusion. In contrast, the axonal compartment demonstrates a remarkable stability in both diffusion and relaxation characteristics. This iron-mediated R2 increase in cell bodies inverts the intrinsic relaxation rate hierarchy from the pattern of the youth (R2_cellbody_ < R2_axon_) to that of the elderly (R2_cellbody_ > R2_axon_), while preserving the consistent ADC ordering (ADC_cellbody_ > ADC_axon_). Consequently, the product term (ADC_cellbody_ − ADC_axon_) × (R2_cellbody_ − R2_axon_) undergoes a sign inversion from negative to positive, leading to the observed transition from positive to negative coupling. Age-related demyelination, which would tend to lower axonal R2, could modulate but is unlikely to solely invert this polarity, supporting the contribution of iron-related changes. This coupling mechanism explains why regions with higher iron content (putamen, globus pallidus and substantia nigra) showed more pronounced coupling shift than iron-sparse areas like the caudate. The regional differences highlight the importance of considering tissue heterogeneity when interpreting MRI data of basal ganglia. Quantification of the continuous coupling transition in these regions will likely lead to a more comprehensive understanding of age-related tissue microstructural changes.

Although our findings demonstrate a significant correlation between diffusion-relaxation coupling metrics and R2_b = 0_ values (a surrogate for tissue properties including iron content), we acknowledge that these coupling parameters are not intended to replace established iron quantification methods like R2* or QSM (Haacke et al., 2004, 2015; Langkammer et al., 2010; Y. Wang & Liu, 2015). Rather, the unique value of diffusion-relaxation coupling lies in providing a complementary dimension of contrast. While mean R2_b = 0_ showed robust age correlations across all regions (Fig. 5C), the coupling metrics revealed a regionally specific decoupling that was most pronounced in iron-rich nuclei. This suggests that diffusion weighting modulates the relaxation contrast in a manner sensitive to specific microstructural configurations altered by aging, such as the differential accumulation of iron in cellular compartments. Thus, our approach contextualizes relaxation changes within a diffusion-weighted framework, potentially offering increased specificity to certain tissue alterations. This complementary information may be particularly valuable for understanding the pathophysiological impact of iron dysregulation in neurodegeneration (Kell, 2009), where the cellular and subcellular distribution of iron may be more clinically relevant than the total iron content alone.

Building upon the relationship between microstructural alterations and diffusion-relaxation coupling patterns, our findings further reveal distinct microstructural signatures between neuronal cell bodies and axonal tracts within the basal ganglia. This compartment-sensitive differentiation offers advantages over conventional diffusion or relaxation metrics alone and may provide a novel avenue to investigating neurodegenerative processes characterized by selective cellular vulnerability. While our current results illustrate this proof-of-concept in aging, the approach shows particular promise for studying conditions like Parkinson’s disease, where selective degeneration of dopaminergic neuron occurs (Z. D. Zhou et al., 2023). Future studies are needed to validate the sensitivity of these coupling metrics to early pathological changes and their potential for monitoring disease progression.

While our study focused on microstructural changes potentially linked to iron, the potential influence of basal ganglia calcification warrants consideration, particularly in elderly populations. The exclusion of severe calcification in our methodology (as specified in Methods) was deliberate, as calcific deposits may introduce competing microstructural effects that could theoretically reverse the iron-driven negative coupling pattern. In calcified regions, neuronal loss and microstructural disintegration typically lead to increased free water content, which has higher ADC and lower R2 values compared with brain tissue (Oyama et al., 2021). Concurrently, calcium’s diamagnetic properties reduce R2 relaxation rates, potentially creating a positive coupling signature (increased ADC with TE, rising R2 with b-value) that could mask iron’s paramagnetic effects. This phenomenon might explain inconsistent coupling patterns across ages, particularly in populations with undetected mild calcification. Our observation that putamen, globus pallidus, and substantia nigra maintained the significant negative coupling (p < 0.01) despite being prone to calcification suggests microstructural changes associated with iron deposition remain the dominant factor in the enrolled 57 healthy participants. Future studies incorporating CT imaging and/or QSM could systematically evaluate how calcification burden modifies the iron-coupling relationship across different age groups.

While our findings are promising, several technical considerations must be addressed to fully realize the potential of diffusion-relaxation coupling. First, our protocol employed only three b-values and three TEs in order to achieve a practically acceptable scan time. The limited sampling precludes robust fitting of more complex models, such as direct inverse Laplace transforms to estimate full diffusion-relaxation distributions. The use of mono-exponential fitting, as a result of limited b-values and TEs, may overlook intravoxel incoherent motion (IVIM) effects at low b-values. We recognize that the analysis of diffusion-relaxation coupling also requires robust fitting algorithms to accurately estimate ADC and R2 values across a range of b-values and TEs. This can be particularly challenging in regions of low signal-to-noise ratio and/or with significant susceptibility artifacts. Emerging computational approaches, including deep learning-based parameter estimation and quality control, may help address these analysis challenges while improving reproducibility (Barbieri et al., 2024; Eidex et al., 2025). The acquisition of multi-b-value diffusion (Dan et al., 2024) and multi-echo R2 (Slator et al., 2021) data requires careful optimization to balance scan time and image quality. Advanced imaging techniques, such as simultaneous multi-slice imaging (Barth et al., 2016; Breuer et al., 2005) or time-division multiplexing EPI (Ji et al., 2021), could be employed to improve efficiency. Furthermore, while comprehensive diffusion-relaxometry sequences like ZEBRA or EPTI exist (Andersson & Sotiropoulos, 2016; Dong et al., 2025; Wongkornchaovalit et al., 2025), our SMT-DWI protocol was designed as a clinically practical modification of a standard vendor sequence, prioritizing high in-plane resolution for deep nuclei imaging and immediate deployment on clinical scanners. Second, to maintain an adequate SNR, a relatively thick slice (4 mm) was chosen for reliable fitting at high b-values and long TEs, despite the increased susceptibility to partial volume effects. Third, our analysis assumes a linear coupling between diffusion and relaxation, providing a simple and interpretable approach. While suitable for an initial investigation, this linear framework cannot capture the full complexity of the diffusion-relaxation correlation space. More advanced non-parametric approaches (Johnson et al., 2024; Manninen et al., 2024; Narvaez et al., 2022; Ning, 2025; Slator et al., 2021, 2023) can recover continuous diffusion-relaxation distributions without imposing a priori compartmental assumptions, offering greater specificity to underlying tissue microstructure. The conceptual model assumes compartmentalized behaviors (e.g., distinct signatures for cell bodies versus axons) that may oversimplify the complex microstructure of brain tissues, particularly in disease states where additional pathological processes (e.g., inflammation, edema, or gliosis) could influence both diffusion and relaxation properties (Slator et al., 2021). Fourth, the current analysis relies on R2 rather than the more specific R2* for iron quantification, which could limit the specificity of our iron-related interpretations since R2 is sensitive to both iron content and other tissue properties, including water content and macromolecular composition (Langkammer et al., 2010). More advanced methods, such as χ-separation (Shin et al., 2021), are needed to distinguish iron from other contributions, including demyelination, to the observed relaxation changes. Lastly, future studies should explore the relationship between diffusion-relaxation coupling and other imaging modalities, such as myelin water imaging (Mackay et al., 1994) and functional MRI (Kwong et al., 1992; Ogawa et al., 1990). Integrating these techniques could provide a more comprehensive understanding of brain tissue microstructures and their alterations in brain development, aging, and pathologic processes.

## 5 Conclusion

In conclusion, our findings demonstrate that age-dependent diffusion-relaxation coupling in the basal ganglia provides a novel approach for probing microstructural changes during brain aging. This technique capitalizes on fundamental biophysical differences between neuronal cell bodies and axonal tracts to detect subtle tissue alterations. The observed transition from positive coupling to negative coupling strongly correlated with regional microstructural alterations, consistent with age-related processes such as iron deposition, though our study cannot definitively isolate iron as the sole contributor, suggesting this method may complement other neuroimaging biomarkers. With continued methodological refinement and clinical validation, diffusion-relaxation coupling may establish itself as a clinically useful tool for investigating brain health and disease mechanisms.

## Data Availability

The sample data and analysis code that support the findings will be available upon reasonable request to the corresponding author.

## References

[IMAG.a.1195-b1] Alexander, G. (1986). Parallel organization of functionally segregated circuits linking basal ganglia and cortex. Annual Review of Neuroscience, 9(1), 357–381. 10.1146/annurev.neuro.9.1.357 3085570

[IMAG.a.1195-b2] Andersson, J. L. R., & Sotiropoulos, S. N. (2016). An integrated approach to correction for off-resonance effects and subject movement in diffusion MR imaging. NeuroImage, 125, 1063–1078. 10.1016/j.neuroimage.2015.10.019 26481672 PMC4692656

[IMAG.a.1195-b3] Angelova, D. M., & Brown, D. R. (2015). Iron, aging, and neurodegeneration. Metals, 5(4), 2070–2092. 10.3390/met5042070

[IMAG.a.1195-b4] Barbieri, M., Hooijmans, M. T., Moulin, K., Cork, T. E., Ennis, D. B., Gold, G. E., Kogan, F., & Mazzoli, V. (2024). A deep learning approach for fast muscle water T2 mapping with subject specific fat T2 calibration from multi-spin-echo acquisitions. Scientific Reports, 14(1), 1–11. 10.1038/s41598-024-58812-2 38589478 PMC11002020

[IMAG.a.1195-b5] Barth, M., Breuer, F., Koopmans, P. J., Norris, D. G., & Poser, B. A. (2016). Simultaneous multislice (SMS) imaging techniques. Magnetic Resonance in Medicine, 75(1), 63–81. 10.1002/mrm.25897 26308571 PMC4915494

[IMAG.a.1195-b6] Beard, J. (2003). Iron deficiency alters brain development and functioning. Journal of Nutrition, 133, 1468–1472. 10.1093/jn/133.5.1468S 12730445

[IMAG.a.1195-b7] Beaulieu, C. (2002). The basis of anisotropic water diffusion in the nervous system—A technical review. NMR in Biomedicine, 15, 435–455. 10.1002/nbm.782 12489094

[IMAG.a.1195-b8] Breuer, F. A., Blaimer, M., Heidemann, R. M., Mueller, M. F., Griswold, M. A., & Jakob, P. M. (2005). Controlled aliasing in parallel imaging results in higher acceleration (CAIPIRINHA) for multi-slice imaging. Magnetic Resonance in Medicine, 53(3), 684–691. 10.1002/mrm.20401 15723404

[IMAG.a.1195-b9] Chakravarthy, V. S., Joseph, D., & Bapi, R. S. (2010). What do the basal ganglia do? A modeling perspective. Biological Cybernetics, 103(3), 237–253. 10.1007/s00422-010-0401-y 20644953

[IMAG.a.1195-b10] Chatterjee, A., Bourne, R. M., Wang, S., Devaraj, A., Gallan, A. J., Antic, T., Karczmar, G. S., & Oto, A. (2018). Diagnosis of prostate cancer with noninvasive estimation of prostate tissue composition by using hybrid multidimensional MR imaging: A feasibility study. Radiology, 287(3), 864–873. 10.1148/radiol.2018171130 29393821 PMC5978456

[IMAG.a.1195-b11] Connor, J. R., Menzies, S. L., St. Martin, S., & Mufson, E. J. (1990). Cellular distribution of transferrin, ferritin and iron in the human brain. Journal of Neuroscience Research, 27, 595–611. 10.1002/jnr.490270421 2079720

[IMAG.a.1195-b12] Dan, G., Sun, K., Luo, Q., & Zhou, X. J. (2024). Single-Shot Multi-b-value (SSMb) diffusion-weighted MRI using spin echo and stimulated echoes with variable flip angles. NMR in Biomedicine, 37(12), e5261. 10.1002/nbm.5261 39308034 PMC12270497

[IMAG.a.1195-b13] Does, M. D., & Gore, J. C. (2000). Compartmental study of diffusion and relaxation measured in vivo in normal and ischemic rat brain and trigeminal nerve. Magnetic Resonance in Medicine, 43(6), 837–844. 10.1002/1522-2594(200006)43:6<837::AID-MRM9>3.0.CO;2-O 10861878

[IMAG.a.1195-b14] Dong, Z., Reese, T. G., Lee, H., Huang, S. Y., Polimeni, J. R., Wald, L. L., & Wang, F. (2025). EPTI for SNR-efficient distortion-free in-vivo mesoscale diffusion MRI and microstructure imaging. Magnetic Resonance in Medicine, 93, 1535–1555. 10.1002/mrm.30365 39552568 PMC11782731

[IMAG.a.1195-b15] Du, Y. P., Zhou, X. J., & Bernstein, M. A. (2002). Correction of concomitant magnetic field-induced image artifacts in nonaxial echo-planar imaging. Magnetic Resonance in Medicine, 48(3), 509–515. 10.1002/mrm.10249 12210916

[IMAG.a.1195-b16] Eidex, Z., Safari, M., Wynne, J., Qiu, R. L. J., Wang, T., Viar Hernandez, D., Shu, H.-K., Mao, H., & Yang, X. (2025). Deep learning based apparent diffusion coefficient map generation from multi-parametric MR images for patients with diffuse gliomas. Medical Physics, 52(2), 847–855. 10.1002/mp.17509 39514841 PMC11788019

[IMAG.a.1195-b17] Finkelstein, A., Cao, X., Liao, C., Schifitto, G., & Zhong, J. (2022). Diffusion encoding methods in MRI: Perspectives and challenges. Investigative Magnetic Resonance Imaging, 26, 208–219. 10.13104/imri.2022.26.4.208

[IMAG.a.1195-b18] Gold, G., Kövari, E., Herrmann, F. R., Canuto, A., Hof, P. R., Michel, J. P., Bouras, C., & Giannakopoulos, P. (2005). Cognitive consequences of thalamic, basal ganglia, and deep white matter lacunes in brain aging and dementia. Stroke, 36(6), 1184–1188. 10.1161/01.STR.0000166052.89772.b5 15891000

[IMAG.a.1195-b19] Haacke, E. M., Cheng, N. Y. C., House, M. J., Liu, Q., Neelavalli, J., Ogg, R. J., Khan, A., Ayaz, M., Kirsch, W., & Obenaus, A. (2005). Imaging iron stores in the brain using magnetic resonance imaging. Magnetic Resonance Imaging, 23(1), 1–25. 10.1016/j.mri.2004.10.001 15733784

[IMAG.a.1195-b20] Haacke, E. M., Liu, S., Buch, S., Zheng, W., Wu, D., & Ye, Y. (2015). Quantitative susceptibility mapping: Current status and future directions. Magnetic Resonance Imaging, 33(1), 1–25. 10.1016/j.mri.2014.09.004 25267705

[IMAG.a.1195-b21] Haacke, E. M., Xu, Y., Cheng, Y. C. N., & Reichenbach, J. R. (2004). Susceptibility weighted imaging (SWI). Magnetic Resonance in Medicine, 52(3), 612–618. 10.1002/mrm.20198 15334582

[IMAG.a.1195-b22] Haber, S. N. (2016). Corticostriatal circuitry. Dialogues in Clinical Neuroscience, 18(1), 7–21. 10.31887/DCNS.2016.18.1/shaber 27069376 PMC4826773

[IMAG.a.1195-b23] Helenius, J., Soinne, L., Perkiö, J., Salonen, O., Kangasmäki, A., Kaste, M., Carano, R. A. D., Aronen, H. J., & Tatlisumak, T. (2002). Diffusion-weighted MR imaging in normal human brains in various age groups. American Journal of Neuroradiology, 23(2), 194–199. 10.1080/j.1600-0455.2003.00104.x 11847041 PMC7975251

[IMAG.a.1195-b24] Ji, Y., Gagoski, B., Hoge, W. S., Rathi, Y., & Ning, L. (2021). Accelerated diffusion and relaxation- diffusion MRI using division multiplexing EPI. Magnetic Resonance in Medicine, 86(5), 2528–2541. 10.1002/mrm.28894 34196032

[IMAG.a.1195-b25] Johansson, J., Lagerstrand, K., Björkman-Burtscher, I. M., Laesser, M., Hebelka, H., & Maier, S. E. (2024). Normal brain and brain tumor ADC: Changes resulting from variation of diffusion time and/or echo time in pulsed-gradient spin echo diffusion imaging. Investigative Radiology, 59(10), 727–736. 10.1097/RLI.0000000000001081 38587357 PMC11460738

[IMAG.a.1195-b26] Johnson, J. T. E., Yang, Y., Laun, F. B., Ross, T. J., Topgaard, D., Benjamini, D., & Martin, J. (2024). In vivo disentanglement of diffusion frequency-dependence, tensor shape, and relaxation using multidimensional MRI. Human Brain Mappping, 45, e26697. 10.1002/hbm.26697 PMC11082920 38726888

[IMAG.a.1195-b27] Ke, Y., & Qian, Z. M. (2007). Brain iron metabolism: Neurobiology and neurochemistry. Progress in Neurobiology, 83(3), 149–173. 10.1016/j.pneurobio.2007.07.009 17870230

[IMAG.a.1195-b28] Kell, D. B. (2009). Iron behaving badly: Inappropriate iron chelation as a major contributor to the aetiology of vascular and other progressive inflammatory and degenerative diseases. BMC Medical Genomics, 2, 1–79. 10.1186/1755-8794-2-2 19133145 PMC2672098

[IMAG.a.1195-b29] Kwong, K. K., Belliveau, J. W., Chesler, D. A., Goldberg, I. E., Weisskoff, R. M., Poncelet, B. P., Kennedy, D. N., Hoppel, B. E., Cohen, M. S., Turner, R., Cheng -, H. M., Brady, T. J., & Rosen, B. R. (1992). Dynamic magnetic resonance imaging of human brain activity during primary sensory stimulation. Proceedings of the National Academy of Sciences of the United States of America, 89(12), 5675–5679. 10.1073/pnas.89.12.5675 1608978 PMC49355

[IMAG.a.1195-b30] Langkammer, C., Krebs, N., Goessler, W., Scheurer, E., Ebner, F., Yen, K., Fazekas, F., & Ropele, S. (2010). Quantitative MR imaging of brain iron: A postmortem validation study. Radiology, 257(2), 455–462. 10.1148/radiol.10100495 20843991

[IMAG.a.1195-b31] Mackay, A., Whittall, K., Adler, J., Li, D., Paty, D., & Graeb, D. (1994). In vivo visualization of myelin water in brain by magnetic resonance. Magnetic Resonance in Medicine, 31(6), 673–677. 10.1002/mrm.1910310614 8057820

[IMAG.a.1195-b32] Manninen, E., Bao, S., Landman, B. A., Yang, Y., Topgaard, D., & Benjamini, D. (2024). Variability of multidimensional diffusion-relaxation MRI estimates in the human brain. Imaging Neuroscience, 2, imag-2-00387. 10.1162/imag_a_00387 PMC12315732 40800299

[IMAG.a.1195-b33] Moos, T., Nielsen, T. R., Skjørringe, T., & Morgan, E. H. (2007). Iron trafficking inside the brain. Journal of Neurochemistry, 103(5), 1730–1740. 10.1111/j.1471-4159.2007.04976.x 17953660

[IMAG.a.1195-b34] Mori, S., & Zijl, P. C. M. Van. (1995). Diffusion weighting by the trace of the diffusion tensor within a single scan. Magnetic Resonance in Medicine, 33(1), 41–52. 10.1002/mrm.1910330107 7891534

[IMAG.a.1195-b35] Narvaez, O., Svenningsson, L., Yon, M., Sierra, A., & Topgaard, D. (2022). Massively multidimensional diffusion-relaxation correlation MRI. Frontiers in Physics, 9, 793966. 10.3389/fphy.2021.793966

[IMAG.a.1195-b36] Ning, L. (2025). Maximum-entropy and subspace methods for high-resolution relaxation-diffusion distribution estimation. Imaging Neuroscience, *3*, IMAG.a.113. 10.1162/IMAG.a.113 PMC12365690 40843023

[IMAG.a.1195-b37] Ning, L., Gagoski, B., Szczepankiewicz, F., Westin, C. F., & Rathi, Y. (2020). Joint RElaxation-diffusion imaging moments to probe neurite microstructure. IEEE Transactions on Medical Imaging, 39(3), 668–677. 10.1109/TMI.2019.2933982 31398113 PMC7164590

[IMAG.a.1195-b38] Ogawa, S., Lee, T., Kay, A., & Tank, D. (1990). Brain magnetic resonance imaging with contrast dependent on blood oxygenation. Proceedings of the National Academy of Sciences of the United States of America, 87, 9868–9872. 10.1073/pnas.87.24.9868 2124706 PMC55275

[IMAG.a.1195-b39] Oyama, J., Yokoyama, K., Fujioka, T., Nariai, T., Karakama, J., Maehara, T., & Tateishi, U. (2021). Incidental T2 hyperintensities in the medial part of the bilateral globus pallidus are possibly an age-related physiological finding. Neuroradiology Journal, 34(6), 575–584. 10.1177/19714009211014129 33949230 PMC8649193

[IMAG.a.1195-b40] Pfefferbaum, A., Adalsteinsson, E., Rohlfing, T., & Sullivan, E. V. (2008). Diffusion tensor imaging of deep gray matter brain structures: Effects of age and iron concentration. Neurobiol Aging, 31(3), 482–493. 10.1016/j.neurobiolaging.2008.04.013.Diffusion 18513834 PMC2815127

[IMAG.a.1195-b41] Rodriguez-Sabate, C., Morales, I., & Rodriguez, M. (2022). The influence of aging on the functional connectivity of the human basal ganglia. Frontiers in Aging Neuroscience, 13, 785666. 10.3389/fnagi.2021.785666 35095470 PMC8789673

[IMAG.a.1195-b42] Sener, R. N. (2001). Diffusion MRI: Apparent diffusion coefficient (ADC) values in the normal brain and a classification of brain disorders based on ADC values. Computerized Medical Imaging and Graphics, 25(4), 299–326. 10.1016/S0895-6111(00)00083-5 11356324

[IMAG.a.1195-b43] Shin, H., Lee, J., Hyun, Y., Ho, S., Jang, J., Oh, S., Nam, Y., Jung, S., Kim, S., Fukunaga, M., Kim, W., Jin, H., & Lee, J. (2021). x-separation: Magnetic susceptibility source separation toward iron and myelin mapping in the brain. NeruoImage, 240, 118371. 10.1016/j.neuroimage.2021.118371 34242783

[IMAG.a.1195-b44] Slator, P. J., Cromb, D., Jackson, L. H., Ho, A., Counsell, S. J., Story, L., Chappell, L. C., Rutherford, M., Hajnal, J. V, Hutter, J., & Alexander, D. C. (2023). Non-invasive mapping of human placenta microenvironments throughout pregnancy with diffusion-relaxation MRI. Placenta, 144, 29–37. 10.1016/j.placenta.2023.11.00237952367

[IMAG.a.1195-b45] Slator, P. J., Hutter, J., Palombo, M., Jackson, L. H., Ho, A., Panagiotaki, E., Chappell, L. C., Rutherford, M. A., Hajnal, J. V., & Alexander, D. C. (2019). Combined diffusion-relaxometry MRI to identify dysfunction in the human placenta. Magnetic Resonance in Medicine, 82(1), 95–106. 10.1002/mrm.27733 30883915 PMC6519240

[IMAG.a.1195-b46] Slator, P. J., Palombo, M., Miller, K. L., Westin, C. F., Laun, F., Kim, D., Haldar, J. P., Benjamini, D., Lemberskiy, G., de Almeida Martins, J. P., & Hutter, J. (2021). Combined diffusion-relaxometry microstructure imaging: Current status and future prospects. Magnetic Resonance in Medicine, 86, 2987–3011. 10.1002/mrm.28963 34411331 PMC8568657

[IMAG.a.1195-b47] Sowell, E. R., Peterson, B. S., Thompson, P. M., Welcome, S. E., Henkenius, A. L., & Toga, A. W. (2003). Mapping cortical change across the human life span. Nature Neuroscience, 6(3), 309–315. 10.1038/nn1008 12548289

[IMAG.a.1195-b48] Stanisz, G. J., Odrobina, E. E., Pun, J., Escaravage, M., Graham, S. J., Bronskill, M. J., & Henkelman, R. M. (2005). T1, T2 relaxation and magnetization transfer in tissue at 3T. Magnetic Resonance in Medicine, 54(3), 507–512. 10.1002/mrm.20605 16086319

[IMAG.a.1195-b49] Sun, K., Dan, G., Zhong, Z., & Zhou, X. J. (2023). Multi-readout DWI with a reduced FOV for studying the coupling between diffusion and T 2 relaxation in the prostate. Magnetic Resonance in Medicine, 4086, 250–258. 10.1002/mrm.29636 PMC12233207 36932652

[IMAG.a.1195-b50] Wang, S., Peng, Y., Medved, M., Yousuf, A. N., Ivancevic, M. K., Karademir, I., Jiang, Y., Antic, T., Sammet, S., Oto, A., & Karczmar, G. S. (2014). Hybrid multidimensional T2 and diffusion-weighted MRI for prostate cancer detection. Journal of Magnetic Resonance Imaging, 39(4), 781–788. 10.1002/jmri.24212 23908146 PMC4251798

[IMAG.a.1195-b51] Wang, Y., & Liu, T. (2015). Quantitative susceptibility mapping (QSM): Decoding MRI data for a tissue magnetic biomarker. Magnetic Resonance in Medicine, 73(1), 82–101. 10.1002/mrm.25358 25044035 PMC4297605

[IMAG.a.1195-b52] Ward, R. J., Zucca, F. A., Duyn, J. H., Crichton, R. R., & Zecca, L. (2014). The role of iron in brain ageing and neurodegenerative disorders. Lancet Neurol., 13(10), 1045–1060. 10.1016/S1474-4422(14)70117-6 25231526 PMC5672917

[IMAG.a.1195-b53] Wong, E. C., Cox, R. W., & Song, A. W. (1995). Optimized isotropic diffusion weighting. Magnetic Resonance in Medicine, 34(2), 139–143. 10.1002/mrm.1910340202 7476070

[IMAG.a.1195-b54] Wongkornchaovalit, P., Shao, B., Li, L., Chen, Y., & He, H. (2025). Multi-TE Diffusion MRI dataset for exploring combined diffusion-relaxometry methods in microstructure imaging. Scientific Data, 12(1), 1191. 10.1038/s41597-025-05544-1 40640197 PMC12246157

[IMAG.a.1195-b55] Yablonskiy, D. A., & Haacke, E. M. (1994). Theory of NMR signal behavior in inhomogeneous tissues: The static dephasing regime. Magnetic Resonance in Medicine, 32(4), 749–763. 10.1002/mrm.1910320610 7869897

[IMAG.a.1195-b56] Yoshiura, T., Wu, O., Zaheer, A., Reese, T. G., & Gregory Sorensen, A. (2001). Highly diffusion-sensitized MRI of brain: Dissociation of gray and white matter. Magnetic Resonance in Medicine, 45(5), 734–740. 10.1002/mrm.1100 11323798

[IMAG.a.1195-b57] Zecca, L., Youdim, M. B. H., Riederer, P., Connor, J. R., & Crichton, R. R. (2004). Iron, brain ageing and neurodegenerative disorders. Nature Reviews Neuroscience, 5(11), 863–873. 10.1038/nrn1537 15496864

[IMAG.a.1195-b58] Zhou, X. J., Du, Y. P., Bernstein, M. A., Reynolds, H. G., Maier, J. K., & Polzin, J. A. (1998). Concomitant magnetic-field-induced artifacts in axial echo planar imaging. Magnetic Resonance in Medicine, 39(4), 596–605. 10.1002/mrm.1910390413 9543422

[IMAG.a.1195-b59] Zhou, X. J., Tan, S. G., & Bernstein, M. A. (1998). Artifacts induced by concomitant magnetic field in fast spin-echo imaging. Magnetic Resonance in Medicine, 40(4), 582–591. 10.1002/mrm.1910400411 9771575

[IMAG.a.1195-b60] Zhou, Z. D., Yi, L. X., Wang, D. Q., Lim, T. M., & Tan, E. K. (2023). Role of dopamine in the pathophysiology of Parkinson’s disease. Translational Neurodegeneration, 12(44), 1–15. 10.1186/s40035-023-00378-6 37718439 PMC10506345

